# Effects of exercise prescribed at different levels of claudication pain on walking performance in patients with intermittent claudication: a protocol for a randomised controlled trial

**DOI:** 10.1177/17539447221108817

**Published:** 2022-06-28

**Authors:** Stefan T. Birkett, Jonathan Sinclair, Sally A. Seed, Sean Pymer, Edward Caldow, Lee Ingle, Amy E. Harwood, Anselm Egun

**Affiliations:** School of Sport and Health Sciences, University of Central Lancashire, Preston PR1 2HE, UK; School of Sport and Health Sciences, University of Central Lancashire, Preston, UK; School of Sport and Health Sciences, University of Central Lancashire, Preston, UK; Academic Vascular Surgical Unit, Hull York Medical School, Hull, UK; School of Health and Society, University of Salford, Salford, UK; Department of Sport, Health and Exercise Science, University of Hull, Hull, UK; Centre for Sport, Exercise and Life Sciences, Coventry University, Coventry, UK; Lancashire Teaching Hospitals NHS Foundation Trust, Preston, UK

**Keywords:** pain, peripheral artery disease, supervised exercise programme

## Abstract

**Background:**

Peripheral artery disease affects over 236 million people globally and the classic symptom is intermittent claudication (IC) which is associated with reduction in physical activity. The evidence that supervised exercise programmes (SEPs) improve pain-free and maximal walking distance is irrefutable. However, adherence rates are low with exercise-related pain cited as a contributing factor. National and international guidelines recommend exercising at a moderate to maximal level of claudication pain to improve walking ability; however, exercising pain-free or at mild claudication pain has been shown to achieve this outcome. There is limited evidence that compares the relative effects of exercise prescribed at different levels of claudication pain.

**Objective:**

The objective of this study is to directly compare the effects of exercise prescribed at three different levels of claudication pain on walking performance.

**Design:**

This study will be a single-centre randomised controlled trial.

**Methods:**

Based on an *a priori* power calculation, 51 patients with IC will be allocated to 24 weeks of twice-weekly pain-free (PF), moderate pain (MOD-P) or maximal pain (MAX-P) exercise. The PF group will cease exercise at the onset of claudication (1 on the 0–4 IC rating scale), the MOD-P group will stop once moderate pain is reached (2 on the rating scale) and the MAX-P group will stop once maximal pain is reached (4 on the rating scale).

**Analysis:**

Outcome measures will be assessed at baseline, 12 and 24 weeks adopting an analysis of covariance (ANCOVA) to compare MWD across three time points. The primary outcome for the trial will be change in maximal treadmill walking distance at 12 and 24 weeks.

**Registration:**

Trial registration number: NCT04370327.

## Introduction

Peripheral artery disease (PAD) is characterised by atherosclerotic lesions of the arteries in the lower limbs, resulting in a reduction in blood flow.^1^ Globally, it is estimated that 236 million people are living with PAD, with the number of cases increasing.^2^ A classic symptom of PAD is intermittent claudication (IC), characterised by ischemic muscle pain in the leg precipitated by exertion and relieved by rest.^3,4^ PAD is associated with various comorbidities such as diabetes mellitus, hypertension and dyslipidaemia, as well as reductions in physical function, quality of life and balance.^3,5,6^ National and international guidelines^4,7^ recommend exercise therapy as the first-line treatment for patients with IC, generally advocating 2 h per week of a supervised exercise over a 3-month period, with patients being encouraged to exercise to the point of moderate or maximal pain. Exercise therapy, via supervised exercise programmes (SEPs), is supported by high-quality evidence for its clinical and cost-effectiveness, costing less than a tenth of angioplasty.^8,9^

Despite the plethora of evidence demonstrating the benefits of SEPs, less than half of the vascular units in the United Kingdom have access to one and patient uptake rates are low.^10,11^ One of the primary reasons for poor adherence may be the level of pain prescribed during SEPs. Indeed, exercise-induced pain is a major barrier to physical activity in this population,^12^ and the level of pain prescribed during SEPs influences completion rates.^13^ When exercise is prescribed at higher levels of pain, completion rates are lower. However, current evidence and guidelines advocate exercising at moderate to maximal pain,^7,14–16^ despite evidence to the contrary, suggesting that mild- or pain-free exercise improves walking ability.^17–20^ The lack of adequately powered, randomised clinical trials investigating the effects of exercise prescribed at differing levels of claudication pain has also been highlighted in a recent scientific statement from the American Heart Association.^16^ As such, it remains unclear which level of claudication is optimal for improving walking performance in patients with IC.^21^ The aim of this trial is to directly compare the effects of exercise prescribed at different levels of claudication [pain-free (PF), moderate pain (MOD-P) and maximal pain (MAX-P)] on (1) maximal and pain-free walking distance; (2) adherence; (3) acceptability, tolerability and enjoyment of the exercise intervention; (4) walking behaviour and physical activity; and (5) barriers and quality of life. It is cautiously expected that maximal pain will lead to greatest improvements in walking performance.

## Methods and analysis

This study is a single-centre randomised controlled trial. Participants will be randomly allocated to 24 weeks of PF, MOD-P or MAX-P exercise with outcomes measured at baseline (visit 1), 12 weeks (visit 2) and 24 weeks (visit 3). All sessions will be supervised by a qualified exercise professional within an existing community pathway. As this programme duration is longer than suggested in current UK guidelines, outcomes will be measured at 12 and 24 weeks to ensure generalisability of the findings to UK SEPs.

### Setting

The trial will be conducted in one centre under the Heartbeat Northwest Cardiovascular Prevention and Rehabilitation charity programme. Patients will attend an SEP from the choice of three Heartbeat cardiovascular rehabilitation sites across Lancashire, UK (Preston, Chorley and Blackpool). Testing will also be conducted at these locations.

### Study registration

The trial was prospectively registered on ClinicalTrials.gov (NCT04370327). Any amendments required to this protocol will seek approvals from the research ethics committee before implementation and will be fully reported in the final trial report.

### Participants

Patients recently diagnosed with IC by a vascular surgeon or vascular specialist nurse will be referred to the SEP and screened for study participation.

### Inclusion criteria

1. >18 years old;
2. Resting ankle brachial pressure index (ABPI) <0.9;
3. Able to walk unaided;
4. English speaking and able to follow exercise instructions;
5. Able to provide informed consent.

### Exclusion criteria

1. Those who have critical limb-threatening ischaemia (rest pain and/or tissue loss);
2. Those undergoing active cancer treatment;
3. Those presenting with any significant comorbidities or contraindications to exercise testing or training in accordance with the American College of Sports Medicine;^22^
4. Unstable/uncontrolled coronary heart disease.

### Study procedures

**Figure 1.**
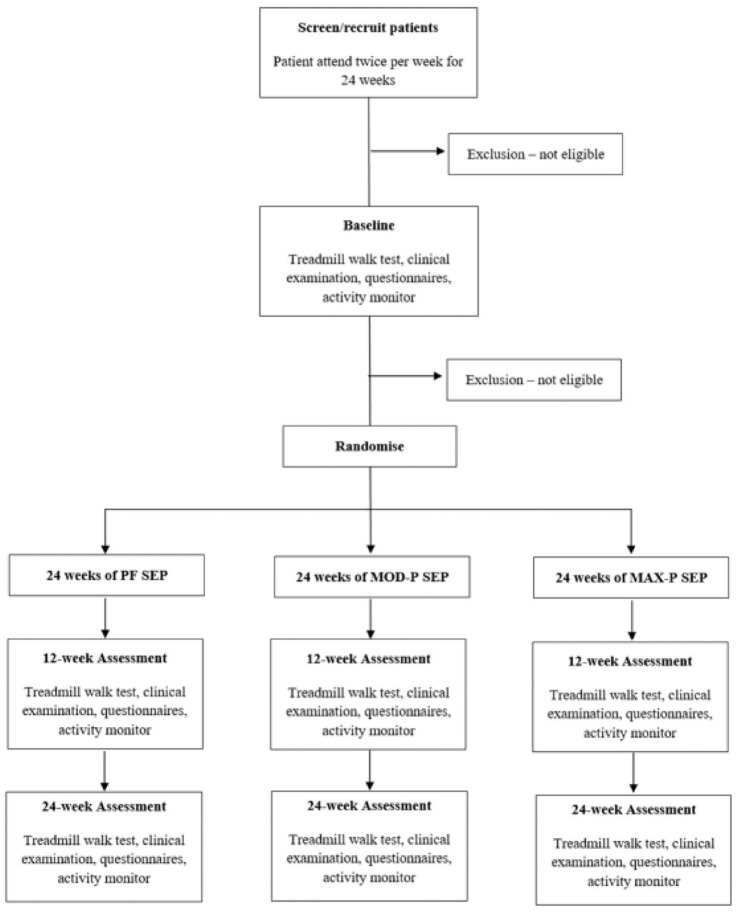
Study flow chart.

An outline of the participant pathway for the study is presented in Figure 1. Once referred, patients will be contacted by a member of the research team. Eligible patients will be offered the opportunity to participate in this study and receive a participant information sheet. A subsequent phone call (at least 48 h later) from the research team will confirm those who wish to participate. Informed consent will be obtained at the baseline assessment visit. Baseline procedures will include: a clinical examination, a treadmill walking test, anthropometrics, barriers to physical activity and quality-of-life questionnaires. Patients will also be asked to wear a physical activity monitor for a duration of 7 days following the baseline assessment. Eligible participants will subsequently be randomised to 24 weeks of twice-weekly PF, MOD-P or MAX-P SEP. All measures completed at baseline will be repeated at 12 and 24 weeks. Semi-structured interviews will be conducted at 24 weeks to evaluate patient acceptability, tolerability and enjoyment. Those who decline study participation will still be offered SEP participation as part of usual care.

### Intervention

**Figure 2.**
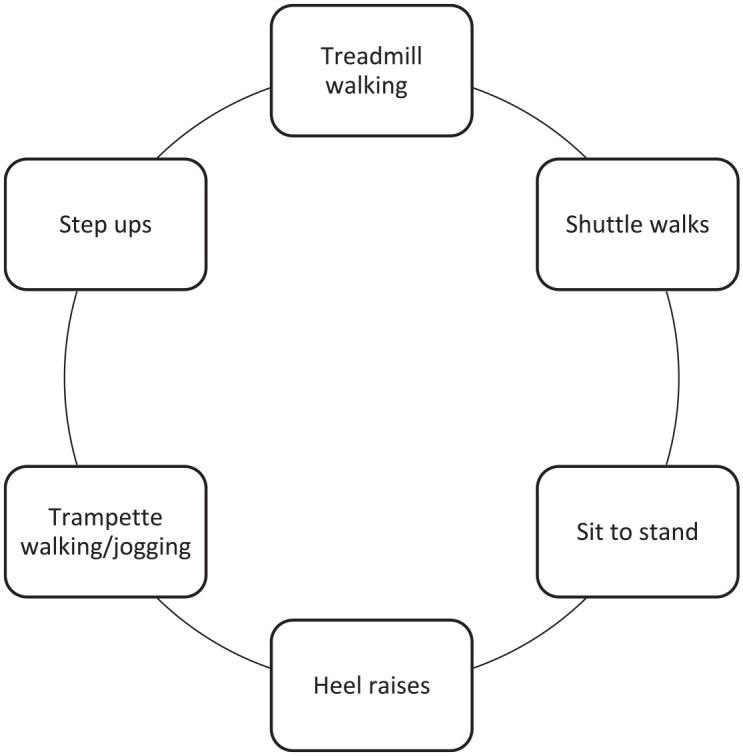
Visual representation of the PAD-specific exercise circuit.

**Figure 3.**
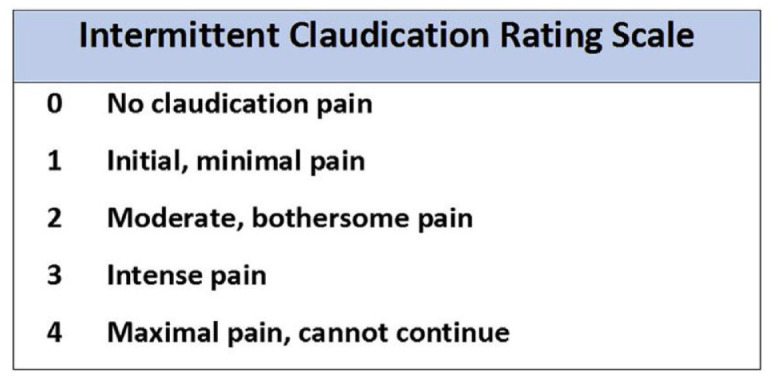
The Intermittent Claudication rating scale which will be used by the patients to grade claudication pain during the exercise intervention. Taken from AACPVR Guidelines for Cardiac Rehabilitation and Secondary Prevention Programs (2013).

The SEP will consist of a circuit^23^ (Figure 2) lasting for 60 min, including a 10-min warm-up and cooldown.^24^ Participants will be individually prescribed exercise until they achieve the desired rating of claudication pain on each station within 3–5 min. They will start the next exercise once the pain has subsided. The American Association of Cardiovascular and Pulmonary Rehabilitation (AACVPR) 0–4 scale to rate claudication pain will be adopted (Figure 3). Participants in the PF group will cease exercise at the onset of claudication (1 on the rating scale), the MOD-P group will stop once moderate pain is achieved (2 on the rating scale) and the MAX-P group will stop once maximal pain is achieved (4 on the rating scale). Participants who are unable/unwilling to comply with the protocols (achieve desired rating of claudication pain) will be permitted to cease involvement and continue with usual-care SEP. To be regarded as having sufficiently adhered to the treatment protocol, patients must complete a minimum of 80% of sessions over 24 weeks (38 of 48).

### Randomisation and blinding

The random allocation sequence will be generated by the trial statistician on a 1:1 basis using a computer program random number generator. To ensure allocation concealment, researchers will request randomisation from the principal investigator on completion of all baseline assessments using a sealed, opaque, sequentially numbered envelope. All outcome assessors will be blinded as will the trial statistician. Exercise professionals delivering the interventions cannot be blinded; however, they will not be involved in data analysis or reporting.

### Outcome measures

The primary outcome measure is change in maximal walking distance (MWD) at 12 and 24 weeks. Secondary outcomes include (1) pain-free walking distance (PFWD); (2) adherence; (3) acceptability, tolerability and enjoyment of the exercise interventions; (4) walking behaviour and physical activity; and (5) barriers to physical activity and quality of life.

### Outcome assessments

Clinical examination will include a review of past medical history and current medications, height, weight and cardiovascular risk factor assessment, that is, resting blood pressure and smoking status. Walking behaviour and physical activity will be recorded over a 7-day period, at baseline (1 week prior to commencing the SEP) and at 12 weeks and 24 weeks using an ActiGraph GT9X link activity monitor (ActiGraph, Pensacola, FL, USA). A valid wear time is defined as 4>days of >10 h of wear. Periods of >60 min of consecutive zero reading will be considered as non-wear time. Activity intensities will be assigned adopting cut points on those validated in coronary artery disease populations,^25^ with light, moderate and vigorous classified as <1800 counts/min, 1800–3799 counts/min and >3800 counts/min, respectively. Sedentary bouts will be defined as periods of wear time exceeding 60 min at <150 counts/min.

A graded treadmill walking test will be performed to determine MWD and PFWD. The treadmill protocol will consist of a constant speed of 3.2 km/h and incremental gradient beginning at 0% increasing 2% every 2 min, for a maximum of 15 min^26^ and will be conducted in accordance with published recommendations for implementation, ensuring standardisation.^27^ PFWD will be recorded at the point at which the patient first indicates the onset of claudication pain. MWD will be recorded when the patient can no longer continue due to claudication pain.

Adherence and compliance will be determined by recording the number of training sessions attended and successfully completed in accordance with the exercise protocol. Drop-out from the SEP will also be documented for all study groups in addition to the reason for drop-out, where provided voluntarily by participants.

Quality-of-life measures will be recorded using disease-specific questionnaires. The King’s College Vascular Quality of Life, a 25-item questionnaire with five domains (pain, symptoms, activities, social and emotional), and the Walking Impairment Questionnaire (WIQ) will be used to assess the perceived impact of claudication.^28,29^ Personal and environmental barriers to physical activity in PAD will be based on the studies by Barbosa *et al.*^12^ and Cornelis *et al.*^30^ A 5-point ordinal scale (never, seldom, sometimes, frequently, always) will be used to assess the limiting character of each barrier (see Table 1).^30^ To verify the safety of the interventions performed in the SEP, adverse and serious adverse events will be carefully monitored, recorded and reported in line with the principles of Good Clinical Practice (GCP). Semi-structured interviews adopting a predetermined set of open topics will qualitatively evaluate acceptability, tolerability and enjoyment of the exercise intervention in all groups

**Table 1.** Personal and environmental barriers.

Personal	Environmental
Pain on exertion	Obstacles aggravating pain
Need for rest	Unfavourable weather
Fear of falling	Poor quality or dangerous side walks
Lack of knowledge regarding exercise benefits	No place to rest when experiencing pain
Need for supervision/control	Shortness of space to exercise or be physically active
Lack of time	Hilly terrain
Other health issues	
Financial reasons	

### Sample size

Power analysis performed in G*Power^31^ showed that 39 patients (total) would be needed to attain statistical significance. Based on previously published data investigating a UK-based SEP that adopted a similar exercise circuit^32^ and converting the interquartile range to standard deviation,^33^ a median change in MWD of 143 m and a pooled standard deviation of 111.3 m was calculated. A power of 90% and a significance level of 5% were assumed. A drop-out of approximately 30% will be allowed, yielding a required sample size of 51 patients (17 per group) to be randomised.

### Data collection and management

The protocol and subsequent trial will adhere to the Standard Protocol Items: Recommendations for Clinical Trials (SPIRIT) and adopt the SPIRIT checklist.^34^ Trial data will be collected on a case report form by the research team at baseline, 12 and 24 weeks. The anonymised data will be stored using a password-protected file on the University of Central Lancashire staff OneDrive system and processed using an institutional Microsoft Surface Pro. All electronic data will be anonymised and identifiable via a number only. Paper data, that is, consent forms, will be kept in a locked filing cabinet in the principal investigator’s office for a duration of 5 years after study completion.

### Data analysis

The primary endpoint for the statistical analysis is the change in MWD from baseline to 12 and 24 weeks. An analysis of covariance (ANCOVA) will be used to compare MWD across three time points with baseline MWD as a covariate. Post hoc analysis for the main effects and interactions will be assessed using a Bonferroni adjustment. Group differences will be compared using simple main effects. Secondary outcomes such as PFWD distance will be evaluated adopting the same approaches. Qualitative data will be analysed using inductive thematic analysis whereby themes are identified from the transcripts.^35^

Data will be entered into SPSS (IBM, New York, USA) by a single investigator who will maintain the overall responsibility for data quality. The primary and secondary outcome analyses will be conducted at the conventional (two-sided) 5% alpha level. Where parametric data distribution allows, partial eta squared values will also be reported. To reduce the risk of false-positive claims, secondary analyses will be considered exploratory if non-significant results are obtained from the primary analysis. All analyses will be performed on an intention-to-treat basis. All data will be summarised and reported in accordance with the Consolidated Standards of Reporting Trials (CONSORT) guideline, the template for intervention description and replication (TIDieR) and recently published recommendations.^36–38^

### Patient and public involvement

The study protocol has been discussed with a patient member who is willing to remain a part of the team for the duration of the study and will be invited to attend all trial steering committee meetings. We also aim to hold three to four PPI meetings over the course of the study to aid with addressing potential recruitment or retention issues and aid with dissemination of the study findings.
